# Integrated optimisation for production capacity, raw material ordering and production planning under time and quantity uncertainties based on two case studies

**DOI:** 10.1007/s12351-020-00609-y

**Published:** 2020-10-03

**Authors:** Wei Xu, Dong-Ping Song

**Affiliations:** 1 Material System Co., Ltd., Shanghai, China; 2 School of Management, University of Liverpool, Chatham Street, Liverpool, L69 7ZH UK

**Keywords:** Multi-stage supply chain, Raw material ordering and production planning, Capacity planning, Uncertainties, Case study, Genetic algorithms

## Abstract

This paper develops a supply chain (SC) model by integrating raw material ordering and production planning, and production capacity decisions based upon two case studies in manufacturing firms. Multiple types of uncertainties are considered; including: time-related uncertainty (that exists in lead-time and delay) and quantity-related uncertainty (that exists in information and material flows). The SC model consists of several sub-models, which are first formulated mathematically. Simulation (simulation-based stochastic approximation) and genetic algorithm tools are then developed to evaluate several non-parameterised strategies and optimise two parameterised strategies. Experiments are conducted to contrast these strategies, quantify their relative performance, and illustrate the value of information and the impact of uncertainties. These case studies provide useful insights into understanding to what degree the integrated planning model including production capacity decisions could benefit economically in different scenarios, which types of data should be shared, and how these data could be utilised to achieve a better SC system. This study provides insights for small and middle-sized firm management to make better decisions regarding production capacity issues with respect to external uncertainty and/or disruptions; e.g. trade wars and pandemics.

## Introduction

In the Supply Chain (SC) context, a wide range of decisions could influence Supply Chain Performance (SCP); e.g. management of material inputs and outputs, production and transport planning, coordination among SC facilities, demand forecasting, and information management. To establish a fully collaborative decision-making mechanism that benefits the whole SC, as well as each member is a complex and challenging process. Managing Raw Materials (RMs) ordering and production planning ensures companies having required materials to build or produce a product with lower cost (cost is accrued at the point of acquisition and is listed as a current asset on a company’s balance sheet). Production capacity limits the income when the product is in high demand, but increases the potential cost during times of low demand. Integrated decisions are especially complicated and difficult when the SC faces disruption (e.g. trade war or natural disaster). Thus, it is important to use best practice for managing RM inventory and production with an integrated consideration of production capacity.

The majority of SCs involve physical products, often at their core, and face a variety of uncertainties. Those uncertainties include: (1) Uncertainty related to the focal company, i.e., internal organisation uncertainty e.g. product characteristics, manufacturing process and control, and decision complexity (2) Uncertainty that is within the realm of control of the focal company or its SC partners, and (3) External uncertainties from factors outside the SC, which are outside a company’s direct span of control (Simangunsong et al. 2012).

It is difficult for companies to manage SC uncertainty; especially small and mid-sized companies. These firms lack expertise in the context of trade wars (e.g. China versus USA) and natural disasters (e.g. Covid-19 pandemic). Consequently, their SCs are more vulnerable. However, these companies contribute to the SCs of large companies. Problems for SMEs not only negatively impact the economy, but also the large companies that rely on them as partners.

To address uncertainty issues in SC networks, is complicated due to the substantial number of combinations of uncertainties. However, real case studies provide deeper insights into those impacts. In fact, the production planning process in uncertain situations has been considered in a variety of contexts (e.g. Mula et al. 2006; Liu et al. 2011; Huang et al. 2014; Mardan et al. 2015; Jeon and Kim 2016; Govindan and Cheng 2018; Zhao and You 2019). Production capacity has been studied in terms of SC planning, constraints (Chen and Xiao 2015), relation to SC risks (Jain and Hazra, 2017) and location and capacity (De Rosa et al. 2014). However, production capacity is a possible issue or risk when SCs face disruption (Hariharan et al. 2020).

This paper models the integrated planning and control for dynamic material flows. This includes RM ordering and Finished Goods (FG) production in the presence of multiple types of uncertainties that exist in the processes of: RM procurement and delivery, FG production and remanufacturing, shipment distribution, and customer demand arrivals. The production capacity decision is also considered and optimised along with integrated RM ordering and production decisions. The study supports SC decision-making in three ways: (1) Managing of material procurement and production is a key component of the SC decision-making framework; (2) Production capacity decisions relating to SC disruptions (e.g. trade war and pandemics) provides insights to managers facing similar issues; (3) The model considers actual case studies and quantifies the benefits of integrated planning in various uncertain conditions; (4) Uncertainties include the dimensions of (a) time (e.g. lead-time and delay) and (b) quantity (e.g. demand, order, supply, defects that occur in information and material flows). Thereby providing a better understanding of to what degree integrated planning offers economic benefits in different scenarios. The cases offer insights into which specific types of data should be shared and how these data could be utilised to achieve an integrated SC system.

The paper is organised as follows: Sect. 2 provides a literature review of production and inventory management models in uncertain situations. Section 3 develops a SC model based on the two case studies through mapping the SCs and identifying and classifying the existing uncertainties in each SC. Section 4 presents a mathematical model for describing and managing the SC. Section 5 discusses the model solution and offers practical strategies. A Stochastic approximation algorithm and a Genetic Algorithm (GA) are developed to optimise some of the parameterised strategies. In Sect. 6, experiments are performed on one of the companies to quantify and compare the strategies including the company's original strategy in a range of scenarios. Finally, Conclusions are offered.

## Literature review

Uncertainty is an inherent characteristic of most SCs. SC uncertainty includes: late delivery, damage and loss, product demand, inaccurate order information, order cancellations, exchange rates, transportation times, market pricing, operation yield uncertainty, production lead time, quality uncertainty, machine breakdowns, human error, absenteeism, and changes to product structure (Davis 1993; Mula et al. 2006; Blackhurst et al. 2007; Snyder et al. 2016; Yue and You 2016). Micro-level uncertainty, Meso-level uncertainty and Macro-level uncertainty are discussed by Flynn et al. (2016). Uncertainty may be classified into two broad categories: lead time and quantity.

The literature on modelling production and inventory management in uncertain situations is rich. Mula et al. (2006) review the literature for production planning models under uncertainty. Their focus is on mid-term tactical models for real-world applications. They classify models into four categories: conceptual, analytical, artificial intelligence-based, and simulation. ManMohan and Christopher (2009) provide a survey on modelling SC planning under demand uncertainty using stochastic programming. Govindan and Cheng (2018) edited a special issue to address SC planning problems (such as sustainability assessment, risk mitigation, vendor selection, and SC coordination) in various uncertain situations focusing on applications of stochastic programming and robust optimisation techniques.

For optimal dynamic control policies in production and inventory systems under uncertainty, many researchers consider multi-stage systems with stochastic demand and deterministic lead-time; e.g.: Clark and Scarf (1960), Chen and Zheng (1994), Chen (2000), Chao and Zhou (2009), Fattahi et al. (2018) and Zhang et al. (2019). Bassok and Akella (1991) consider the optimal production level and order quantity problem under supply quality and demand uncertainty. When two or more types of uncertainty (mainly demand and lead-time uncertainties) are modelled, the optimal production control and inventory replenishment policies are often investigated within a single-stage (Song and Zipkin 1996), two-stage (Berman and Kim 2001; He et al. 2002; Yang 2004), or three-stage system (Song and Dinwoodie 2008; Song 2009; 2013). Quality and demand uncertainty are considered for joint procurement and production decisions in a hybrid remanufacturing system (Mukhopadhyay and Ma 2009). Uncertainty on demand, manufacturing and sales-effort cost are considered by Chen et al. (2017). Haji et al. (2011) focus on the optimisation of a specific type of control policies in a two-level inventory system with uncertain demand and lead-time. Dillon et al. (2017) study a two-stage stochastic programming model for inventory management in the blood SC. The optimal base-stock policy is obtained by analysing the steady-state distributions of the system. Jamalnia and Feili (2013) apply a hybrid discrete event simulation and system dynamics method to simulate aggregate production planning that is able to handle uncertainties in demand, supply, and production. Hammami et al. (2014) develop a scenario-based stochastic model for supplier selection and purchased quantity decision under uncertain currency exchange rates and price discounts. Bi-objective optimisation for multiple-stage SCs with the consideration of international and domestic market has been considered (Roe et al. 2015). Pasandideh et al. (2015) focus on bi-objective optimisation of a multi-product multi-period three-echelon supply-chain-network with stochastic demand, production time, and set-up time. Gholamian et al. (2015) consider multi-product multi-site production planning in a SC with demand uncertainty. Mardan et al. (2015) present an integrated emergency ordering and production planning model for multi-item, multi-product production planning with demand and supply uncertainty. Modak and Kelle (2019) examine inventory management in the context of a dual-channel (retail and online) SC under price and delivery-time dependent stochastic customer demand. Shafiq and Savino (2019) focus on a manufacturer’s capacity procurement decisions with demand and RM procurement lead time uncertainty.

Production capacity has been considered recently in relation to: (1) optimal order quantity and production capacity in centralised and decentralised settings (Glock et al. 2020), (2) multi-echelon SC model involving different production/storage capacities, bio-refineries technologies, and transportation modes (Gilani and Sahebi 2020), (3) product replenishment orders and production capacity in a two-stage stochastic approach study (Ben Abid et al. 2020), and (4) production capacity as a constraint in SC modelling (Arasteh 2020).

Modelling techniques used in the SC risk literature include: stochastic dynamic programming (Clark and Scarf 1960; Song and Zipkin 1996; Chen 2000; Berman and Kim 2001; He et al. 2002,2019; Yang 2004; Song and Dinwoodie 2008; Chao and Zhou 2009; Song 2009, 2013; Quddus, Chowdhury et al. 2018; Salehi et al. 2019), steady state distribution (Chen and Zheng 1994; Haji et al. 2011), convex programming with Lagrange multiplier (Bassok and Akella 1991), probability analysis with first-order condition (Mukhopadhyay and Ma 2009) simulation-based optimisation (Song 2013; Roe et al. 2015), hybrid simulation (Jamalnia and Feili 2013), mixed integer scenario-based stochastic programming (Hammami et al. 2014), stochastic mixed integer linear programming (Pasandideh et al. 2015), multi-objective mixed-integer non-linear programming (Gholamian et al. 2015), two-stage decision-making (Mardan et al. 2015), Mixed Integer Non-Linear Programming (MINLP) (Keyvanshokooh et al. 2016; Yue and You 2016; Mousavi et al. 2019). The use of dynamic programming for seeking optimal dynamic control policies is appropriate because the underlying systems are less complicated and analytically tractable. For more complex systems, with many products and multiple uncertainties, the analytical approach is intractable and is often replaced with artificial intelligence and simulation-based methods (Mula et al. 2006; Song 2013) . Snyder et al. (2016) discuss common modelling approaches. Govindan et al. (2017) summarise the existing optimisation techniques for dealing with uncertainty such as recourse-based stochastic programming, risk-averse stochastic programming, robust optimisation, and fuzzy mathematical programming—mathematical modelling and solution approaches.

Further concerns about SC disruption (Bode and Wagner 2015; Chopra and Sodhi 2004; Christopher and Lee 2004; Craighead et al. 2007; Dixit et al. 2020; Dolgui et al. 2018; Fahimnia et al. 2015; Heckmann et al. 2015; Hendricks and Singhal 2005; Kleindorfer and Saad 2005; Li and Zobel 2020; Manuj and Mentzer 2008; Snyder et al. 2016; Tang 2006; Tomlin 2006) have been raised. Production capacity is one of the risks. Studies on integrated ordering, production, and production capacity decisions are rare; especially on actual cases. There is also a lack of consideration of the integrated operational processes between functional SC members (e.g. supplier, manufacturer, warehousing, transportation, and customer) in the presence of multiple uncertainties. This paper contributes by considering: (1) How to model SC operations with multiple uncertainties from a systems perspective (considering all behaviours, interactions and relationships in the system); and (2) How in the face of multiple uncertainties to improve decisions on integrated production and RM ordering, and production capacity. This paper extends earlier work (Roe et al. 2015) by focusing on the application of SC modelling to: (1) SCs for small and medium sized firms; (2) provide simpler and more effective decision making; (3) assist companies operating within a domestic marketplace in the face of external disruptions (trade wars, natural disasters and pandemics); (4) evaluated and optimise integrated RM ordering, production, and production capacity; and (5) the use of two separate optimisation methods on decision variables. Table 1 compares this study with other relevant literature in terms of research scopes and methods.

**Table 1:** Comparative table with relevant literature

Most related literature	Material ordering	Production planning	Production capacity planning	SC integration	Real case study	Lead-time uncertainty	Delay uncertainty	Demand uncertainty	Production uncertainty	Delivery uncertainty	Optimisation methods
Bassok and Akella (1991)	X	X	–	X	–	X	–	X	–	–	Lagrange multiplier
Berman and Kim (2001)	X	–	–	X	–	X	–	X	–	–	Markov decision
Yang (2004)	X	X	–	X	–	X	–	X	–	–	Dynamic programming
Song (2009)	X	X	–	X	–	X	–	X	X	–	Markov decision
Gholamian et al. (2015)	X	X	–	X	–	–	–	X	–	–	Mixed-integer programming
Yue and You (2016)	X	X	–	X	X	X	–	X	X	–	Stochastic robust optimization
Fattahi et al. (2018)	–	X	X	X	–	–	–	X	–	–	Multi-stage stochastic programming
Zhao and You (2019)	X	X	X	X	X	–	–	X	X	–	Two‐stage robust fractional programming
Gilani and Sahebi (2020)	–	X	X	X	–	–	–	X	X	–	Robust optimization
Ben Abid et al. (2020)	X	X	–	X	X	–	–	X	X	–	Two-stage stochastic programming
Roe et al. (2015)	X	X	–	X	X	X	X	X	X	X	GA
This paper	X	X	X	X	X	X	X	X	X	X	GA; Stochastic approximation

## Model development from case studies

Two medium-sized manufacturers in China are considered. These companies are representative as their SCs include multiple functions and entities: multiple suppliers, manufacturing, private warehouses, transportation companies, and many customers. Case company A is an aluminum producer with 900 employees located in Shandong province in China. They produce four alloys of aluminum (A199.90, A199.85, A199.70A and A199.70) sold domestically in China. Three of the RMs are purchased competitively from a group of suppliers. The fourth major input is electrical power sole sourced and supplied continuously. Therefore, only three main RM suppliers need to be considered. Case company B is chemical producer with 150 employees located in Jiangxi province in China. This sino-foreign joint-venture produces fine chemicals, pharmaceutical intermediates, pesticide intermediates and dye intermediates. It had annual sales of 10 million pounds sterling the year data was supplied (2010). In summary, the Cases involve 3 main Suppliers with FG supplying many other companies. Case B's SC is more complicated due to special requirements on RM storage and transportation.

**Fig. 1:**
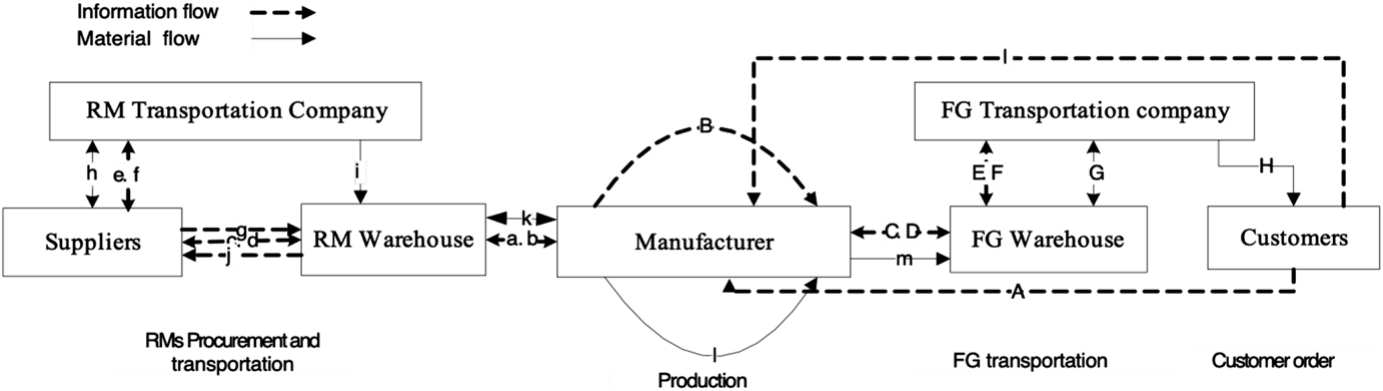
Generalised SC model of information and material flows-based on the two cases. ( Adapted from Roe et al. 2015, p. 88)

The SC structure in the two companies are similar in terms functional activities, information and material flows and associated uncertainties. However, the scale and scope of uncertainties differ. Primary data has been collected through multiple methods; including: group and individual interviews and non-participative observation. Due to confidentiality, the data was exported directly from the case companies’ ERP system for the period from end of 2009 and early 2010. The delay in release of data was deemed necessary due to the competitive nature of the business. In summary, both cases involve manufacturers with multiple final products and multiple main RMs with multiple suppliers for each RM. A generalised and simplified SC model of information and material flows for the two cases is shown in Fig. 1.

The SC model consists of two major processes: (1) RM ordering and transportation, and (2) FG production, transportation and customer fulfilment. RM ordering and transportation includes the following 13 activities:

- Manufacturer shares the production plan with RM warehouse
- RM warehouse reports the RM on-hand inventory information to manufacturer
- RM warehouse places order to suppliers
- Supplier provides feedback on inventory availability to RM warehouse
- Supplier contacts RM transport company to arrange transfer
- Transport company confirms the transfer requirements with suppliers
- Supplier provides transfer information to RM warehouse
- RM transport company picks up RM from supplier
- RM transport company ships RM to RM warehouse
- RM warehouse confirms receipt to supplier and makes payment for RM received
- RM warehouse updates inventory and delivers RM to manufacturer
- Manufacturer produces FG
- Manufacturer transfers FG to FG warehouse

The second process (FG production, transportation and satisfying customer demand) includes the following nine activities:

A. Customer places order to manufacturer
B. Manufacturer receives order and applies internal checking
C. Manufacturer shares customer order information with FG warehouse
D. FG warehouse reports inventory information to manufacturer
E. FG warehouse contacts FG transport company to arrange transfer
F. Transport company confirms transfer requirements with FG warehouse
G. Transport company picks up FG from FG warehouse
H. Transport company transfers FG to customer
I. Customer confirms receipt and makes payment to manufacturer

The above activities can be further categorised into four sub-models: (1) Customer Order (*A, B, C*); (2) Manufacturing/Production (*a, k, l, m*); (3) RM Ordering and Transportation (*b, c, d, e, f, g, h, i, j*); and (4) FG Customer Fulfilment with Transportation (*D, E, F, G, H, I*) model.

### Uncertainties in the SC

The SC system is subject to various uncertainties. Sub-Model I (customer order) involves quantity uncertainty in customer demand, representing the unpredictable nature of external markets. Other inherent uncertainties are: contracted delivery date, order lead-time, order quantity errors, lead-time of delayed orders (correction of errors in initial orders). Uncertainty ranges vary substantially for the two case companies. For example, the upper bound of customer order information lead-time is around 14 days for Company A and 7 days for company B.

Sub-Model II (manufacturing) uncertainties are related to material flow. While internal information processes may influence performance, internal information uncertainty is addressed as part of production lead-time. Both bounds of production lead-time are impacted by labour working time. Company management information systems (ERP) may be incompatible with the existing production control system and or incompatible with the management information systems of SC partners resulting in information and production uncertainty. Low labour skills influence product quality. Defective products require remanufacture. Remanufacturing lead-time is subject to production plan, production capacity and relevant RM availability—leading to further uncertainty. These uncertainties impact both companies. Finally, FG transfer may be delayed due to FG availability or communication errors.

Sub-Model III (RMs) experiences uncertainty in information flow. Uncertainty is a function of the characteristics of the RM and the supplier relationship. In both cases, the main RM order is placed by email or telephone with suppliers. While the focal firms have ERP systems with supplier management function, suppliers usually remain unintegrated. However, Chinese business culture with its industry-oriented professional organisations builds informal relationships that improve SC relationships. Uncertainty occurs in material flows due to inventory availability, transportation capacity, and traffic congestion. Due to special requirements for transporting chemicals, the lead-time and delay uncertainties are higher for company B.

Sub-Model IV (FGs) uncertainties relate to transportation (similar to Sub-Model III). FG availability depends on FG inventory and the production plan. Customer requirements in FG quality, packaging, and delivery may also cause delays.

In summary, the sources of uncertainties are: (1) information flow, (2) material flow, and (3) customer demand. The uncertainties, they can be classified into three groups: lead-time, quantity, and delay. Table 2 summarises the nature of the uncertainties in the four sub-models.

**Table 2:** Classifications of uncertainties in four sub-models

		Time uncertainty		Quantity uncertainty
		Lead-time	Delay time	
Sub-Model I	Demand	Customer contracted delivery date		Random demand
	Information flow	Customer order information lead-time	Handling delayed/inaccurate order	Inaccurate orders
Sub-Model II	Material flow	Production lead-time	Handling defective products for remanufacturing	Defective products
Sub-Model III	Information flow	RM order information lead-time/booking transportation lead-time	Handling delayed RM order	
	Material flow	RM availability/RM transportation lead-time	Handling delayed RM transportation	Fraction of RM to be delayed
Sub-Model IV	Information flow	Arranging FG transportation lead-time	Handling delayed FG information	
	Material flow	FG availability /FG transportation lead-time	Handling delayed FG transportation	Fraction of shipment to be delayed

### SCM challenges for case companies

There are two main operational modes: (1) Normal mode—domestic and export, and (2) Domestic focus. While acting as a global supplier is the normal mode of operation, at certain times demand and accessibility of foreign markets decline. For example, during times of partner (US/China trade war) or global (pandemic) tension.

The main decisions are: placing RM orders to suppliers and determining production quantity for effective customer fulfilment. These decisions are complex due to the many SC uncertainties (Table 2). Furthermore, any plan to increase ordering of RMs and produce more FGs to improve service levels and avoid backordering, could significantly increase inventory costs. The challenge to management is in determining the most appropriate trade-off.

More recently global trade tension (e.g. between the US and China), present the case companies’ SCs to face decisions on whether to withdraw from foreign markets due to mounting cost. Differences in standards and manufacturing processes between domestic and export markets impact production capacity considerations. That is, capacity for different markets is not directly interchangeable. Both case companies are increasingly focusing on their domestic market. The Covid-19 pandemic is a contributor to this shift in attention. As both companies are based in China, the lockdown initiation and relaxation is out of step with foreign customers. This results in a significant decline in international orders with an unknown recovery timeline. Consequently, a new focus on only the domestic marketplace. Hence, a sudden urgency to re-evaluate the impact of decisions regarding RM procurement, production and production capacity on companies at a time of uncertainty and financial stress. With the increasing discussion of the need for domestic production independence for an increasing range of products, modelling the associated costs is increasingly important to an increasing number of firms in an increasing number of counties.

## Mathematic modelling

Mathematical models representing the four sub-models and the associated uncertainties are now provided. Roe et al. (2015) provided a comprehensive formulation for both domestic and international SC under various uncertainties. However, Roe et al. (2015) focused only on operational decisions of RM ordering and production quantity. This study examines the domestic SC only, but considers two planning levels of decisions: tactical (production capacity) and operational (material ordering and production quantity) decisions. For consistency, the notation in Roe et al. (2015) are followed as closely as possible.

*Input parameters, state variables and intermediary variables*

- *T*: the number of planning time periods;
- *x*_o_(*t*), *x*_*i*_(*t*): the on-hand inventory of FG or RM *i* at period *t*;
- *r*_*i*_: the amount of RM *i* required to produce one unit of FG;
- *l*_*c*_(*t*): the information lead-time of customer placing an order (from customer releasing the order to manufacturer receiving the order) at period *t*;
- *l*_*c*_^*d*^(*t*): the lead-time of handling delayed customer orders at period *t*;
- *l*_*i*_^*p*^(*t*)*:* the (information) lead-time of placing an order of RM *i* from manufacturer to supplier at period *t*;
- *l*_*i*_^*s*^(*t*): the (physical) lead-time of shipping RM *i* from supplier to RM warehouse at period *t*;
- *l*_*i*_(*t*)*:* the sum of *l*_*i*_^*p*^(*t*) and *l*_*i*_^*s*^(*t*);
- *l*_*i*_^*d*^(*t*)*:* the lead-time of processing delayed procurement of RM *i* at period *t*;
- *l*_o_(*t*)*:* the production lead-time of manufacturer producing FG at period *t*;
- *l*_*o*_^*d*^(*t*): the lead-time of handling defective products at period *t* so that they can be reworked afterwards;
- *l*_*o*_^*p*^(*t*): the (information) lead-time of arranging shipping FG from the FG warehouse to the customer;
- *l*_*o*_^*s*^(*t*): the (physical) lead-time of shipping the FG from the FG warehouse to transport company then finally arriving at the customer;
- *l*_*s*_(*t*): the sum of *l*_*o*_^*s*^(*t*) and *l*_*o*_^*p*^(*t*), i.e. the total lead-time of shipping FGs from the FG warehouse to the customer at period *t*;
- *l*_*s*_^*d*^(*t*): the lead-time of processing delayed shipments at period *t* so that they can be shipped afterwards;
- *s*_*l*_(*t*): the contracted lead-time of manufacturer satisfying the customer order at period *t*;
- *ξ*_*d*_(*t*): the random variable representing the ratios of on-time and delayed customer orders received/ processed by manufacturer at period *t;*
- *ξ*_*i*_(*t*): the random variable representing the fraction of RM orders received/ processed by suppliers on time at period *t;*
- *ξ*_*o*_(*t*):the random variable representing the fraction of useable FG produced on time initiated at period *t;*
- *ξ*_*s*_(*t*): the random variable representing the fraction of FG orders received by customer on time at period *t;*
- *d*(*t*): the expected customer demands for FG at period *t*;
- *η*_*d*_(*t*): the random variable representing a rate that perturbs the expected customer demand at period *t;*
- *D*(*t*): equals *d*(*t*)·*η*_*d*_(*t*), representing the random demand of FG during period *t*;
- *D*_*o*_^*r*^(*t*): the on time received customer demand at period *t*;
- *D*_*o*_^*d*^(*t*): the delayed portion of customer demands at period *t*;
- *DMD*(*t*): the actually received customer orders by the manufacturer at period *t* that are ready to fulfil;
- *u*_*i*_^*r*^(*t*): the amount of orders for RM *i* received on time by suppliers at period *t*;
- *u*_*i*_^*d*^(*t*)*:* the delayed amount of orders for RM *i* at period *t*;
- *URM*_*i*_(*t*): the RM warehouse actually received RM *i* at period *t*;
- *u*_*o*_^*r*^(*t*): the FG production requirement at period *t*;
- *u*_*o*_^*s*^(*t*)*:* the FG production ability at period *t*, which has considered the constraints*;*
- *u*_*o*_^*S*^(*t*): the amount of useable FG, whose production is initiated at period *t*;
- *u*_*o*_^*d*^(*t*): the amount of defective FG whose production is initiated at period *t;*
- *UFG*_*o*_(*t*): the amount of useable FG that the manufacturer actually produces at period *t,* which has considered the production lead time*;*
- *s*_*o*_^*r*^(*t*): the amount of FG that could be used to satisfy customer demand at period *t;*
- *s*_*o*_^*R*^(*t*): the FG delivered to customers on time at period *t;*
- *s*_*o*_^*d*^(*t*): the delayed portion of finish goods to customers at period *t;*
- *CFG*_*o*_(*t*): the amount of FG that customer actually receives at period *t;*
- *c*_o_^*h*^, *c*_*i*_^*h*^: the inventory holding cost for per unit of FG, or RM *i*;
- *c*_o_^*b*^: the penalty cost for backordering one unit of FG;
- *c*_*o*_^*p*^: the fixed cost for producing one unit of FG;
- *c*_*o*_^*s*^: the setup cost for producing one unit of FG;
- *c*_*o*_^*d*^: the penalty cost for defective production;
- *c*_*o*_^*t*^, *c*_*i*_^*t*^: the transportation cost for shipping one unit of finish goods, or RM *i*;
- *c*_*or*_^*d*^: the penalty cost for one unit of delayed customer order (due to quantity uncertainty);
- *c*_*f*_^*d*^: the penalty cost for one unit of delayed FG shipment (due to quantity uncertainty);
- *c*_*i*_^*d*^: the penalty cost for one unit of delayed RM (due to quantity uncertainty);
- *c*_*o*_^*m*^: the bank payment commission fee with delay penalty cost.

*Decision variables*

- *u*_*i*_(*t*): the planned order quantity for RM *i* at period *t*, which is an operational decision;
- *u*_*o*_(*t*): the planned production quantity for FG at period *t*, which is an operational decision;
- *U*_*o*_: the maximum production capacity (workforce resource) in one period, which is a tactical decision.

### Customer order model

Customer orders at each period are impacted by uncertainty (Table 2) under Sub-model I. The demand quantity uncertainty level is represented by a random variable *η*_*d*_(*t*). There are two types of dynamic lead time (lead-time of placing order *l*_*c*_(*t*) and lead time of handling delayed order *l*_*c*_^*d*^(*t*)) in the SC. These lead times influence when the customer orders are actually ready to fulfil. The following equations are based on Roe et al. (2015).

1 $$D\left(t\right)=d(t){\eta }_{d}\left(t\right)$$

2 $${D}_{o}^{r}\left(t\right)=D\left(t\right)\cdot {\xi }_{d}\left(t\right)$$

3 $${D}_{o}^{d}\left(t\right)=D\left(t\right)\cdot (1-{\xi }_{d}\left(t\right))$$

4 $$DMD\left(t\right)=\sum_{j=1}^{t}{D}_{o}^{r}(j)\cdot I\left\{j+{l}_{c}\left(j\right)=t\right\}+\sum_{j=1}^{t}{D}_{o}^{d}\left(j\right)\cdot I\left\{j+{l}_{c}\left(j\right)+{l}_{c}^{d}\left(j+{l}_{c}\left(j\right)\right)=t\right\}$$

where *I*{*.*} is an indicator function, it takes 1 if the condition in {} is true; 0, otherwise. Equation (1) represents the customer order with quantity uncertainty. Equation (2) represents the part of customer order that is received by the manufacturer on time at period *t*, where *ξ*_*d*_(*t*) is a random variable to represent the ratio of on-time and delayed customer demand (i.e. the incompleteness of customer order received). Equation (3) represents the delayed portion of customer order at period *t*, which requires additional processing to make it ready to be fulfilled. Equation (4) represents the amount of customer orders that the manufacturer actually receives at period *t* to be fulfilled, which is the sum of on-time received customer orders, *D*_*o*_^*r*^(.), generated at the period in advance of the required customer order information lead-time, *l*_*c*_(.), and the sum of previously delayed customer orders, *D*_*o*_^*d*^(.), which become ready to fulfil at period *t*. There is an extra lead-time *l*_*c*_^*d*^(.), representing the additional time required to handle the delayed portion of the order due to inaccurate order information. This extra lead-time is often random, but may be related to the time of error identification.

### Production Model

The production process follows the production plan *u*_*o*_(*t*) subject to capacity constraints. Quantity uncertainty is mainly caused by defective products. The required production quantity at period *t* includes two parts: the production plan *u*_*o*_(*t*) and the amount requiring rework (*u*_*o*_^*d*^(.))—scheduled at the current period with delay uncertainty accounted for. The following equations are based on Roe et al. (2015), in which Eq. (7) has been adjusted to appropriately reflect the production capacity.

5 $${u}_{o}^{r}\left(t\right)={u}_{o}\left(t\right)+\sum_{j=1}^{t}{u}_{o}^{d}\left(j\right)\cdot I\left\{j+{l}_{o}\left(j\right)+{l}_{o}^{d}(j+{l}_{o}\left(j\right))=t\right\}$$

6 $${u}_{o}^{d}\left(t\right)={u}_{o}^{s}\left(t\right)\left(1-{\xi }_{o}\left(t\right)\right)$$

7 $${u}_{o}^{s}\left(t\right)=min\left\{\left.{U}_{o},{u}_{o}^{r}\left(t\right),{(x}_{i}\left(t\right)+{URM}_{i}\left(t\right))/{r}_{i})\right\}\right.$$

8 $${u}_{o}^{S}\left(t\right)={u}_{o}^{s}\left(t\right)\cdot {\xi }_{o}\left(t\right)$$

9 $${UFG}_{o}\left(t\right)=\sum_{j=1}^{t}{u}_{o}^{S}\left(j\right)\cdot I\left\{j+{l}_{o}\left(j\right)=t\right\}$$

10 $${x}_{o}\left(t+1\right)={x}_{o}\left(t\right)+{UFG}_{o}\left(t\right)-DMD\left(t\right)$$

Equation (5) represents production requirements at period *t*, consisting of planned production *u*_o_(*t*) and the amount of required rework *u*_*o*_^*d*^ (the sum of defective FG to be reworked during this period). The lead-time uncertainty of the delayed activity (i.e. remanufacturing lead-time) *l*_*o*_^*d*^(.), implies that defective products may not be reworked upon detection*.* Equation (6) represents the amount of defective FG production initiated at period *t*, in which (1-*ξ*_*o*_(*t*)) represents the quantity uncertainty level (i.e. rate of production of defective product). Equation (7) represents production at period *t*, subject to the available production capacity (*U*_*o*_(*t*)), the production requirement *u*_*i*_^*r*^(*t*), and RM availability. Where (*x*_i_(*t*) + *URM*_*i*_(*t*))/*r*_*i*_ is the available RM *i* at period *t*, depending on the on-hand inventory *x*_i_(*t*), newly received RM *i* quantity *URM*_*i*_(*t*), and the amount of RM *i* required to produce one unit of FG (*r*_*i*_). Equation (8) represents the useable FG (production initiated at period *t*)*.* Equation (9) represents the useable FG completed during period *t* with a production lead-time *l*_o_(*t*). Equation (10) updates the FG inventory state. The FG inventory level at period *t* + 1 equals the FG on-hand inventory level at period *t*, *x*_o_(*t*), plus the newly completed useable FG (*UFG*_*o*_(*t*) at period *t*), minus the received customer demands *DMD*(*t*) at period *t*.

### RM ordering and shipping model

RM ordering and shipping focuses on RM procurement and RM on-hand inventory updating. The quantity uncertainty is represented by (1 − *ξ*_*i*_(*t*)). The physical and information lead-time uncertainties of shipping RMs are represented by *l*_*i*_^*s*^(*t*) and *l*_*i*_^*p*^(*t*) respectively. The lead-time uncertainty of delayed activity is represented by *l*_*i*_^*d*^(*t*). The following equations are based on Roe et al. (2015).

11 $${u}_{i}^{r}\left(t\right)={u}_{i}\left(t\right)\cdot {\xi }_{i}\left(t\right)$$

12 $${u}_{i}^{d}\left(t\right)={u}_{i}\left(t\right)\cdot {(1-\xi }_{i}\left(t\right))$$

13 $${l}_{i}\left(t\right)={l}_{i}^{p}\left(t\right)+{l}_{i}^{s}\left(t\right)$$

14 $${URM}_{i}\left(t\right)=\sum_{j=1}^{t}{[u}_{i}^{r}\left(j\right)\cdot I\left\{j+{l}_{i}\left(j\right)=t\right\}]+\sum_{j=1}^{t}{[u}_{i}^{d}\left(j\right)\cdot I\{j+{l}_{i}^{d}\left(j\right)+{l}_{i}(j+{l}_{i}^{d}\left(j\right))=t\}]$$

15 $${x}_{i}\left(t+1\right)={x}_{i}(t)+{URM}_{i}\left(t\right)-{u}_{o}^{s}\left(t\right)\cdot {r}_{i}$$

Equation (11) represents the amount of on-time procurement for RM *i*, *u*_*i*_^*r*^(*t*), which is influenced by the procurement plan *u*_*i*_(*t*) and a random variable *ξ*_*i*_(*t*). Equation (12) represents the delayed procurement quantity for RM *i*, where (1 − *ξ*_*i*_(*t*)) represents the quantity uncertainty level (the fraction of RM delayed). Equation (13) represents the total procurement (replenishment) lead-time for RM *i*, *l*_*i*_(*t*) that includes the RM order information lead-time and booking transportation lead-time in the information flow (*l*_*i*_^*s*^(*t*)) and the RM availability and RM transportation lead-time in the material flow (*l*_*i*_^*p*^(*t*)). Equation (14) represents the total RM *i* received by the manufacturer at period *t* taking into account the procurement lead-time *l*_*i*_(*t*) and the delayed RM procurement lead time *l*_*i*_^*d*^(*t*). Equation (15) updates the on-hand inventory state of RM *i*. The RM *i* inventory level at period *t* + 1 is equal to the RM inventory level at period *t*, plus the received RM *i* from suppliers at period *t*, minus the used amount of RM at period *t*.

### Customer fulfilment model

Customer fulfilment focuses on FG satisfying customer demands by transferring goods from FG warehouse to customers. Satisfying customer orders depends on the: size of customer order, FG on-hand inventory level, and useable FG produced by the manufacturer in the period. The quantity uncertainty level is represented by (1 − *ξ*_*s*_(*t*)) to reflect the fraction of FG that has shipping delayed. The information and physical lead-time uncertainties of shipping the FG are represented by *l*_*o*_^*p*^(*t*) and *l*_*o*_^*s*^(*t*), respectively. The lead-time uncertainty of handling delayed shipping is represented by *l*_*s*_^*d*^(*t*). The following equations are based on Roe et al. (2015).

16 $${s}_{o}^{r}\left(t\right)=\mathit{min}\{DMD\left(t\right),{x}_{o}\left(t\right)+U{FG}_{O}\left(t\right)\}\;if\;{x}_{o}\left(t\right)\ge 0$$

17 $${s}_{o}^{r}\left(t\right)=\mathit{min}\{DMD\left(t\right)-{x}_{o}\left(t\right),U{FG}_{O}\left(t\right)\}\;if\;{x}_{o}\left(t\right)<0$$

18 $${s}_{o}^{R}\left(t\right)={s}_{o}^{r}\left(t\right)\cdot {\xi }_{s}\left(t\right)$$

19 $${s}_{o}^{d}\left(t\right)={s}_{o}^{r}\left(t\right)\cdot {(1-\xi }_{s}\left(t\right))$$

20 $${l}_{s}\left(t\right)={l}_{o}^{p}\left(t\right)+{l}_{o}^{s}\left(t\right)$$

21 $${CFG}_{o}\left(t\right)=\sum_{j=1}^{t}{s}_{o}^{R}\left(j\right)\cdot I\left\{j+{l}_{s}(j)=t\right\}+\sum_{j=1}^{t}{s}_{o}^{d}\left(j\right)\cdot I\left\{j+{l}_{s}^{d}\left(j\right)+{l}_{s}(j+{l}_{s}^{d}\left(j\right))=t\right\}$$

Equations (16) and (17) represent the fulfilled customer demand (also called shipment) at period *t* corresponding to situations without backlogged demands (i.e. *x*_*o*_(*t*) ≥ 0) and with backlogged demands (*x*_*o*_(*t*) < 0), respectively*.* Equation (18) represents the amount of shipment released on-time at period *t* to the customer with transportation uncertainty. Equation (19) represents the delayed amount of shipment at period *t*. (1 − *ξ*_*s*_(*t*)) represents the quantity uncertainty level (fraction of FG delayed). Equation (20) represents total shipping lead-time *l*_*s*_(*t*), including the lead-time in the information flow (*l*_*o*_^*s*^(*t*)) to arrange FG transportation and the transportation lead-time in the FG flow (*l*_*o*_^*p*^(*t*)). Equation (21) represents the amount of FG that the customer actually receives in period *t*, which is the sum of the shipments that were released on-time and the delayed shipments that were received in period t.

#### Operational planning decision optimisation

Under a given production capacity *U*_*o*_, the operational planning problem is to find the optimal RM ordering and FG production planning decisions {*u*_*o*_(*t*), *u*_*i*_(*t*)} for the planning horizon. Let *J*_*op*_(*U*_*o*_) denote the optimal expected total operational cost in the SC for the production capacity *U*_*o*_. The operational planning optimisation problem is formulated as:

22 $$\begin{aligned} {J}_{op}({U}_{o})&=minE\sum_{t=1}^{T}\left\{\left[{[D}_{o}^{d}\left(t\right)+\left|d\left(t\right)-DMD\left(t\right)\right|\right]{\cdot c}_{or}^{d}+{s}_{o}^{R}\left(t\right)\cdot {c}_{o}^{t}\right.\\ &\quad +\left[{\left(DMD\left(t\right)-{s}_{o}^{r}\left(t\right)\right)}^{+}+{\left(-{x}_{o}\left(t\right)\right)}^{+}\right]\cdot {c}_{o}^{b}+[{s}_{o}^{d}\left(t\right)+{|CFG}_{o}\left(t\right)-{s}_{o}^{r}\left(t\right)|]{\cdot c}_{f}^{d}\\ &\quad +{CFG}_{o}\left(t\right){\cdot c}_{o}^{m}+{u}_{o}^{s}\left(t\right)\cdot {c}_{o}^{p}+{u}_{o}^{s}\left(t\right)\cdot {c}_{o}^{s}+{u}_{o}^{d}\left(t\right)\cdot {c}_{o}^{d}\\ &\quad +\sum_{i=1}^{I}{x}_{i}\left(t\right)\cdot {c}_{i}^{h}+{x}_{o}^{+}\left(t\right)\cdot {c}_{o}^{h}+\sum_{i=1}^{I}{u}_{i}^{r}\left(t\right)\cdot {c}_{i}^{t}\\ &\quad +\left.\sum_{i=1}^{I}{[u}_{i}^{d}\left(t\right)+|{u}_{i}\left(t\right)-{URM}_{i}\left(t\right)|]\cdot {c}_{i}^{d}+{U}_{o}\cdot {c}_{o}^{cap}\right\}\end{aligned}$$

where [.]^+^ takes the positive value of the expression within the bracket. In Eq. (22), the first term represents the customer order delay and inaccurate quantity penalty cost; the second term represents the FG transportation cost; the third term indicates the FG reverse cost including the current period and the accumulated amount; the fourth term indicates the FG shipping delay and inaccurate quantity penalty cost; the fifth term denotes the payment delay penalty cost and banking fee; the sixth to the eleventh terms represent the production fee, setup cost, defective quality penalty cost, RM inventory holding cost, FG inventory holding cost, and RM transportation cost, respectively; the twelfth term represents the RM transportation delay penalty cost; and the final term represents the production capacity (workforce resource) cost.

#### Tactical planning decision optimisation

The tactical planning problem is to seek the optimal production capacity *U*_*o*_. Let J denote the optimal total SC cost after optimising the production capacity. The tactical planning optimisation problem can be formulated as:

23 $$J=min{J}_{op}({U}_{o})$$

## Model solution and optimisation

The mathematical model is difficult to solve analytically due to its complexity. Simulation and artificial intelligence-based methods are, therefore, appropriate techniques to assess appropriate decisions to optimize SCP. This section presents non-parameterised and parameterised strategies that have been effective and practically applicable (e.g. Chen and Zheng 1994; Chen 2000; Castellano et al. 2018), and then develops a GA optimisation tool to optimise the parameterised strategies.

### Non-parameterised strategies

Strategy I is the company’s original strategy (described in the experiment section).

Strategy II applies lot-for-lot. That is, information sharing and cooperation between customer and the manufacturer in terms of actual demand information. The production plan is based on the customer orders that the manufacturer actually receives. The RM ordering plan depends on the amount of RMs required to produce the planned FG. This strategy can be described as:

$$\begin{aligned} u_{o} \left( t \right) & = {\max}\left\{ {0,DMD\left( t \right)} \right\}; \\ u_{i} \left( t \right) & = u_{o} \left( t \right) \cdot r_{i} ; \\ \end{aligned}$$

Strategy III applies Just-In-Time (JIT). This involves information sharing, cooperation between customer and the manufacturer, and taking into account the FG inventory-on-hand at the FG warehouse and the RM inventory-on-hand at the RM warehouse during the decision-making process. The aim is to achieve zero inventory at each stage. The production plan is determined as customer orders minus the FG on-hand inventory. The RM plan is determined by the production plan, the required amount of RMs per unit of FG, and the on-hand inventory of RMs. This strategy is described as:

$$\begin{aligned} u_{o} \left( t \right) & = {\max}\left\{ {0,DMD\left( t \right) - x_{{\text{o}}} \left( t \right)} \right\}; \\ u_{i} \left( t \right) & = {\max}\left\{ {0,u_{o} \left( t \right) \cdot r_{i} - x_{i} \left( t \right)} \right\}; \\ \end{aligned}$$

Strategy IV applies Vendor Managed Inventory (VMI). This involves customer order and on-hand inventory information being considered for production planning in the same way as in the JIT strategy. In addition, RM ordering, FG inventory information, the production plan and RM on-hand inventory are taken into account. Thus, RM ordering decisions are based on the: production plan, FG inventory-on-hand, and RM inventory-on-hand. The aim is to achieve zero echelon inventory level. Echelon inventory is the sum of all inventories at the downstream entities from the current to final stage. This is referred to as a VMI strategy, because downstream entities’ demand and inventory information is utilized for RM ordering decisions. This strategy is described as:

$$\begin{aligned} & u_{o} \left( t \right) = {\max}\left\{ {0,DMD\left( t \right) - x_{{\text{o}}} \left( t \right)} \right\}; \\ & u_{i} \left( t \right) = {\max}\left\{ {0,u_{o} \left( t \right) \cdot r_{i} - x_{i} \left( t \right) - x_{{\text{o}}} \left( t \right) \cdot r_{i} } \right\}; \\ \end{aligned}$$

### Parameterised strategies

The JIT and VMI strategies implicitly aim to achieve zero local inventory and echelon inventory, respectively. These strategies rely on the dynamic customer demand information to arrange the production and material ordering to chase demand. Because demand may be unstable and information inaccurate, SCP may be undesirable. In the presence of uncertainty, maintaining a certain level of inventory is a reasonable solution to these challenges. Consequently, two new strategies combining JIT (and VMI) strategy with the (s, S) policy to manage RM ordering and production planning are offered. Here, *s* represents the reorder point and *S* represents the order-up-to point. To distinguish these approaches from the JIT and VMI strategies, the parameterized strategies are denoted as P-JIT and P-VMI, respectively. These designations reflect the pre-specification of a set of control parameters.

These strategies utilize *s*_0_ and *S*_0_ to represent the lower and upper parameters to control the FG production planning. If the on-hand FG inventory level falls below *s*_0_ then the manufacturer produces FG to bring the inventory level up to *S*_0_. *S*_*i*_ and *s*_*i*_ represent the upper and lower control parameters for each type of RMs.

The P-JIT strategy is described as:

$$\begin{aligned} & u_{i} \left( t \right) = {\max}\left\{ {0,S_{i} - x_{i} \left( t \right)} \right\},{\text{ if}}\;x_{i} \left( t \right) \le s_{i} ;\;\;{\text{ and}}\;\;u_{i} \left( t \right) = 0, \, \;\;{\text{otherwise}}; \\ & u_{o} \left( t \right) = {\max}\left\{ {0,S_{o} - x_{o} \left( t \right)} \right\},{\text{ if}}\;x_{o} \left( t \right) \le s_{o} ;\;\;{\text{ and}}\;\;u_{o} \left( t \right) = 0, \, \;\;{\text{otherwise}}. \\ \end{aligned}$$

The P-VMI strategy is described as:

$$\begin{aligned} & u_{i} \left( t \right) = {\max}\left\{ {0,S_{i} - x_{i} \left( t \right) - r_{i} \cdot x_{o} \left( t \right)} \right\}, \, \;\;{\text{if}}\;x_{i} \left( t \right) + r_{i} \cdot x_{o} \left( t \right) \le s_{i} ;\;\;u_{i} \left( t \right) = 0, \, \;\;{\text{otherwise}}; \\ & u_{o} \left( t \right) = {\max}\left\{ {0,S_{o} - x_{o} \left( t \right)} \right\}, \, \;\;{\text{if}}\;x_{o} \left( t \right) \le s_{o} ;\;\;{\text{ and}}\;u_{o} \left( t \right) = 0, \, \;\;{\text{otherwise}}. \\ \end{aligned}$$

One of the main advantages of the above parameterised strategies over non-parameterised strategies is that operational decisions do not directly rely on dynamic customer demand information. The SC system builds a certain level of inventory to buffer against uncertainties and dynamic fluctuation. However, the challenge for the parameterised strategies is that some control parameters (e.g. si and Si) need to be pre-determined in order to control each type of inventory. Ordering and production decisions, therefore, depend on the pre-determined control parameters. Under the integrated decision-making mechanism, control parameters should be optimised collaboratively with procurement and production decisions.

In the case of three main types of RMs and one type of FG (e.g. the second case study), there would be eight control parameters representing the low and high bounds of RMs and FG to trigger the ordering and production decisions. Therefore, the control parameters to be optimised can be presented by a vector of si and Si. Analytically optimising these control parameters are intractable in our problem. Two methods are presented to optimise the parameters in the SC: stochastic approximation and GA.

### Stochastic approximation algorithm to optimise parameterised strategies

Stochastic approximation algorithm is a well-developed gradient-based search method to optimise a set of real parameters (Rubinstein 1986; Qi and Song 2012). In this problem, the control parameters are real numbers and thus suitable for stochastic approximation. Let **s**: = (*s*_1_, *s*_2_, …, *s*_*n*_)^T^ be a vector of control parameters to be optimised, and *J*(**s**) denote the objective function depending on the decision variables **s**. In this case, s represents the parameterised strategy and *J*(**s**) represents the SC operational cost under the given parameterised strategy. The standard form of the stochastic approximation is:

24 $${\mathbf{s}}_{{k + {1}}} = {\mathbf{s}}_{k} - \gamma_{k} \cdot \nabla J_{k} ,$$

where **s**_*k*_ is the parameter vector at the beginning of iteration *k*, ∇*J*_*k*_ is an estimator of the gradient ∇*J*(**s**_*k*_), which is defined as ∇*J*(**s**_*k*_): = (∂*J*(**s**_*k*_)/∂*s*_1_, ∂*J*(**s**_*k*_)/∂*s*_1_, …, ∂*J*(**s**_*k*_)/∂*s*_*n*_)^T^, and γ_*k*_ is a positive sequence of step sizes such that (i) it decreases to zero, (ii) the sum of all the sequence { γ_*k*_} is infinite, and (iii) the sum of its squares is bounded. Typically, the harmonic sequence 1/*k* satisfies all above assumptions for γ_*k*_.

When ∇*J*_*k*_ is estimated using a finite difference of the objective functions, the stochastic approximation algorithm is called a Kiefer-Wolfowitz (KW) algorithm (Rubinstein 1986). In this paper, a modified KW-type algorithm is proposed. Noting the objective function is unlikely differentiable to the control parameters due to various *min* and *max* operations in the dynamic system, the right-side finite difference and the left-side finite difference are estimated simultaneously via simulation. If both sides’ finite differences are positive, the corresponding element in ∇*J*_*k*_ is zero. The rationale is that changing the corresponding parameters on either side will not reduce the objective function. If both sides’ finite differences are negative, the corresponding element in ∇*J*_*k*_ is set to encourage the control parameter to move the steeper descending side. The rationale for this adjustment is based on the greedy strategy. In addition, noting that the stochastic approximation algorithm is sensitive to the initial solution, multiple samples with different initial solutions are run.

### GA to optimise parameterised strategies

GA is one of the best known meta-heuristic algorithms able to tackle difficult combinatorial optimisation problems. GA can optimise parameters as either discrete or real numbers. For this problem, the GA solution can be presented by a vector of *s*_*i*_ and *S*_*i*_, which are continuous real numbers. The GA developed here follows the standard GA including: initialisation, evaluation, selection, recombination/crossover, mutation, evaluation, and reproduction. An adjustment step in the GA procedure ensures all solutions are feasible with respect to the constraints and characteristics of the underlying SC system. For example, if a new solution in the GA offspring population exceeds the boundaries, the solution will be amended to the nearest boundary value of the parameter. Further details of the GA procedure are provided in Xu (2013).

## Experiments

This section uses case company B's data to evaluate the model under different strategies. Based on the historical data, the periodic customer demands for FG are given by the following time series (Roe et al. 2015):

$$d\left(t\right)=\left(-0.0000000035*{t}^{4}-0.0000002988*{t}^{3}+0.0003570609*{t}^{2}-0.0408922814*t+2.9582935980\right)$$

In Sects. 6.1 and 6.2, different strategies are evaluated under the company’s current production capacity. Four non-parameterised strategies including the company’s original strategy, lot-for-lot, JIT and VMI strategies are evaluated and compared. Under the non-parameterised strategies, there is no control parameter determined in advance. Ordering and production decisions are made dynamically based on the input data and the information from cooperative SC members (e.g. customer order, RM and FG inventory). Next, two parameterised strategies are optimised and evaluated using the simulation-based stochastic approximation approach and GA. Finally (Sect. 6.3), the production capacity and the parameterised strategies are optimized in an integrated way.

### Non-parameterised strategy evaluation under fixed production capacity

The company’s original strategy—Strategy I—is extracted from case company B’s historical data (daily inventory and replenishment) as an input matrix.

In the simulation, two levels of SC cooperation are considered. To simplify the experiment all lead-times of normal activities and delayed activities in Table 2 are assumed to follow the same distribution. *LT* denotes the lead-time distribution, which has an upper bound and a lower bound of zero. All time uncertainty parameters follow a uniform distribution and vary within the bounds. There are two levels of *LT*. *LT* ~ U(0, 3) has a shorter lead-time and lower uncertainty representing a higher level of SC cooperation. *LT* ~ U(0, 7) has a longer lead-time and higher uncertainty representing a lower level of SC cooperation. This reflects how greater SC cooperation often leads to reduced lead-time and uncertainty. For example, increasing cooperation between SC members adopting Electronic Data Interchange (EDI) speeds up information exchange and reduces information lead-time.

Quantity uncertainty is denoted as *DoU* (Degree of Uncertainty), representing all quantity uncertainty variables (Table 2). All quantity uncertainty variables are assumed to follow uniform distributions (based on interviews) within the lower and upper bound. Two levels of quantity uncertainty are considered: low level ~ U(0, 0.1), and high level ~ U(0, 0.3).

The combination of two levels of SC cooperation and quantity uncertainty give rise to four different scenarios. Total cost (measured in £000) measures the SCP. Table 3 summarizes SC total costs for the strategies and scenarios. Strategies II–IV perform significantly better than the original strategy (Strategy I). One reason that the company’s original strategy performs poorly is that it is extracted from the historical data, which may represent a specific sample. Strategy III (JIT) achieved the lowest costs in both scenarios with high-level lead-time uncertainty, whereas strategy IV (VMI) achieves the lowest cost in both scenarios with low-level lead-time uncertainty. For strategy II to IV, more inventory information is utilized in decision-making. This confirm the benefits of better utilizing available information. However, strategy III outperforms strategy IV in two scenarios indicating that the way additional information is used influences its value. That is, inappropriate utilization of information is disadvantageous.

**Table 3:** total costs under non-parameterised strategies with different lead-time (LT) and quantity uncertainty (DoU)

Strategy	(Low LT, low DoU)	(High LT, low DoU)	(Low LT, high DoU)	(High LT, high DoU)
1. Original strategy	26,116	29,232	31,929	34,684
2. Lot-for-Lot	23,172	26,910	29,066	32,956
3. JIT	17,490	**19,057**	18,013	**19,093**
4. VMI	**17,447**	19,507	**17,631**	20,523

To examine the impact of cooperation on SC performance for the original strategy and three improved non-parameterised strategies, the relative performance is provided (Table 3). The bold numbers in Table 3 indicate the best results in the corresponding column among different strategies. Table 3 shows: (1) increasing cooperation levels to reduce lead-times and their uncertainties significantly improves cost performance for each strategy. (2) The cost of strategy III and IV are very close, but different strategies provide benefit in different situations. For example, with low lead-time uncertainty VMI is preferable to JIT, However, JIT is preferable to VMI with higher lead-time uncertainty.

The impact of Lead-Time (LT) and quantity uncertainty (DoU) on SC performance as a percentage is provided (Table 4). DoU form high to low level and LT from high to low level are examined. Table 4 shows that (1) for all strategies, that reductions in lead-time or quantity uncertainty leads to reduced cost; (2) the overall impact of lead-time and quantity uncertainty reduction under JIT is smaller than in the other three strategies (0.2–8.2%). However, when quantity uncertainty changes from high to low, VMI strategy performs better.

**Table 4:** The impact of lead-time and quantity uncertainty on non-parameterised strategies’ performance

Strategy	Low LT	High LT	Low DoU	High DoU
	DoU: high- > low (%)	DoU: high- > low (%)	LT: high- > low (%)	LT: high- > low (%)
1. Original	18.20	15.70	10.70	7.90
2. Lot-for-Lot	20.30	18.30	13.90	11.80
3. JIT	2.90	0.20	8.20	5.70
4. VMI	1.00	5.00	10.60	14.10

### Parameterised strategy optimisation under fixed production capacity

To implement P-JIT and P-VMI strategies the appropriate values for the control parameters {*s*_*i*_ and *S*_*i*_, *i* = 0, 1, 2, 3} under fixed production capacity *U*_*o*_ need to be determined. The simulation-based stochastic approximation and GA procedures are used to optimise the control parameters. To facilitate the comparison with non-parameterised strategies, experiments on the same four scenarios are undertaken. In the stochastic approximation (StoApp), the iteration number is 20 and the initial solution number is 100. In the GA procedure, a number of parameters are selected based on pilot runs. For each experiment: the population size is 50, the maximum generation number is 200, and the mutation probability is 0.5. The GA is coded using Matlab R2019a and run on a Laptop with 2.40 GHz. Computational times for each optimisation experiment are in Tables 4 and 5.

**Table 5:** total costs and computational time under optimised P-JIT using StoApp and GA

Scenario	Method	CPU(s)	Cost	Orig%	P-JIT%
Low LT, low DoU	StoApp	53.8	16,628	36.3	4.9
	GA	54.6	16,814	35.6	3.9
High LT, low DoU	StoApp	56.3	17,571	39.9	7.8
	GA	50.9	17,517	40.1	8.1
Low LT, high DoU	StoApp	53.8	17,016	46.7	5.5
	GA	51.3	17,217	46.1	4.4
High LT, high DoU	StoApp	56.8	17,991	48.1	5.8
	GA	50.7	18,508	46.6	3.1

Table 5 and 6 show the StoApp versus the GA generations in four scenarios under parameterised (optimised) P-JIT and P-VMI respectively. The fourth column in Table 5 and 6 shows the total cost. The fifth column shows the reduction percentage from the original strategy in Table 3. The sixth column shows the relative cost difference between non-parameterised JIT(VMI) strategy (from Table 3) versus parameterised P-JIT(P-VMI). Optimised results under StopApp and GA are shown in both Table 5 and 6.

**Table 6:** total costs and computational time under optimised P-VMI using StoApp and GA

Scenario	Method	CPU(s)	Cost	Orig%	P-VMI%
Low LT, low DoU	StoApp	54.0	16,681	36.1	4.4
	GA	52.1	16,822	35.6	3.6
High LT, low DoU	StoApp	59.8	17,558	39.9	10.0
	GA	49.8	18,066	38.2	7.4
Low LT, high DoU	StoApp	54.4	17,060	46.6	3.2
	GA	51.2	17,341	45.7	1.6
High LT, high DoU	StoApp	58.8	17,998	48.1	12.3
	GA	50.5	18,246	47.4	11.1

Tables 4 and 6 show that: (1) compared to the original strategy (Table 3), optimised P-JIT and P-VMI strategy reduces cost from 35.6 to 48.1%; (2) compared to original strategy, StoApp and GA provide very similar improvements. However, under the scenario High LT /Low DoU, P-JIT-GA outperforms P-JIT-StoApp; (3) compared to non-parameterised JIT and VMI strategy (Table 3), P-JIT performs 3.1–7.8% better than the non-parameterised JIT; and P-VMI improves 1.6–12.3% than the non-parameterised VMI strategy (Table 3). It is economically beneficial to appropriately design target local inventory levels;

### Integrated optimisation for production capacity and parameterised strategies

In this sub-section, both the production capacity (Uo) and the parameterised strategy {*s*_*i*_ and *S*_*i*_, i = 0, 1, 2, 3} are optimised via simulation-based StoApp and GA. Results for P-JIT (Table 6) and P-VMI (Table 8) are given. Cost reduction percentage from the P-JIT (Column 4, Table 7), relative cost difference between the StoApp and GA (Column 5, Table 7) and the relative cost difference between P-VMI and P-JIT under the optimal production capacity (Column 6, Table 8) are all provided.

**Table 7:** total costs under optimised production capacity and P-JIT using StoApp and GA

Scenario	Method	Cost	P-JIT%	StoApp-GA%
Low LT, low DoU	StoApp	7016	57.8	8.6
	GA	7680	54.3	
High LT, low DoU	StoApp	9554	45.6	− 0.4
	GA	9516	45.7	
Low LT, high DoU	StoApp	8801	48.3	6.1
	GA	9369	45.6	
High LT, high DoU	StoApp	11,278	39.1	2.5
	GA	11,566	35.7

**Table 8:** total costs under optimised production capacity and P-VMI using StoApp and GA

Scenario	Method	Cost	P-VMI%	StoApp-GA%	P-JIT vs. P-VMI (%)
Low LT, low DoU	StoApp	6041	63.8	8.5	14.0
	GA	6604	60.7		13.9
High LT, low DoU	StoApp	7469	57.5	8.8	14.0
	GA	8187	54.7		21.8
Low LT, high DoU	StoApp	6894	59.6	7.4	20.6
	GA	7443	57.1		21.7
High LT, high DoU	StoApp	8162	54.6	8.1	23.2
	GA	8882	51.3		27.6

From Tables 7 and 8, it can be observed that (1) optimising production capacity could reduce total cost by 35–57% for P-JIT and 51–63% for P-VMI, showing the importance of appropriately managing production capacity; (2) It appears that Stochastic Approximation generally performs better than GA in this problem. P-VMI performs 13–27% better than P-JIT across four scenarios (Column 6, Table 8), which indicates that optimised production capacity with P-VMI outperforms optimised production capacity with P-JIT significantly in all scenarios. This could be due to P-VMI utilizing more inventory information than P-JIT, which is beneficial in an integrated optimization environment.

## Conclusions

Based upon two case studies of SMEs in a developing economy, a SC model for integrated RM ordering and production planning, and production capacity in dynamic stochastic situations is developed. Uncertainties are identified and modelled for: information flow, material flow, information delays, material delays, supply, customer demand, production, and product quality. The SC model consists of four mathematically-formulated sub-models: (1) Customer Order, (2) Manufacturing Model, (3) RMs Ordering with Transportation, and (4) FG Customer Fulfilment. Simulation-based stochastic approximation and GAs are applied to evaluate four non-parameterised strategies and optimise two parameterised strategies for combinations (2 × 2) of SC cooperation and uncertainty.

For fixed production capacity the JIT and VMI strategies perform significantly better than the original and lot-for-lot strategies. The benefits of information sharing and coordinated management is verified by the success of JIT and VMI, that utilise dynamic inventory information to make ordering and production decisions. The benefits of simulations to assist in better decision making and cost minimization for SMEs in developing economies is also illustrated. Scenario analysis finds that increasing cooperation (reducing lead-time and uncertainty) reduced cost by 0.2–18.2% under all strategies. Reducing quantity uncertainty offers mixed results. Therefore, SC managers should consider seeking effective strategies to reduce uncertainty and increase cooperation collectively. Optimised parameterised strategies perform better than non-parameterised strategies. P-JIT strategy outperforms JIT by 3.1–8.1%, whereas P-VMI outperforms VMI by 3.2–12.3%. JIT (or P-JIT) has similar performance to VMI (or P-VMI) in all scenarios under the fixed production capacity. VMI utilises more inventory information than JIT, however, this does not necessarily improve the performance. How information is utilized should be considered carefully. As some of the results are not intuitive, an integrated planning tool could be helpful for managers to better understand their SC. In addition, both simulation-based StoApp and GA improve performance under all scenarios and tested-strategies. In most situations, StoApp slightly outperforms GA. Consequently, as long as a suitable optimising tool is selected, the results are helpful.

Under the integrated planning of tactical production capacity and operational decisions (RM ordering and production planning), production capacity decisions significantly impact cost under all scenarios and strategies. Optimisation of production capacity and control parameters (RM ordering and FG production), can reduce total cost by 35.7–63.8%. Interestingly, P-VMI outperforms P-JIT significantly in all scenarios, which indicates utilizing more inventory information (under P-VMI) is more beneficial in a more integrated optimization environment. Hence, production capacity decisions for RM ordering and production are important. Especially, during trade wars and other disruptions. If too much is produced for international markets, domestic production capacity is limited. However, when international customers lack confidence about procuring from abroad, a foreign producer needs to re-consider RM ordering, production, and production capacity. P-JIT and P-VMI significantly reduce cost for either optimisation tool. Therefore, companies can benefit from either optimisation tool applied to P-JIT or P-VMI. Companies should optimise production capacity decisions, RM ordering and production decisions with an optimisation applied to either P-JIT or P-VMI strategy with respect to their SC environments.

This study supports collaborative decision-making for: (1) managing material procurement and production. This model is a first step toward collaborative decision-making; (2) considering a wide range of uncertainties in the SC, and providing guidelines to make collaborative decision-making framework more practical. For example, how specific data related to information and material flows and delays can be incorporated into a model to support collaborative decision-making mechanisms; (3) decision support assisting Small and Medium-sized Enterprises (SMEs) assess the benefits and impacts of changing collaboration strategy which is especially important in the face of external disruption, e.g. trade war or pandemic. When the SC is disrupted by policy or nature disasters, regular orders are either cancelled or postponed. Decisions made based on integrated RM ordering, production and production capacity could significantly help SEMs survive. SMEs are especially vulnerable in these cases as they lack the financial flow, skills and market power of large companies. Past research in JIT, VMI, lot-for-lot and SC coordination consider large organisations as the focal firm, but SMEs are overlooked. This research assists SME management teams understand the benefits, impacts and requirements of SC collaboration. This study illustrates the latent profit opportunities existing in current manufacturers. Such untapped resources are especially important in less favourable periods: increased competition and economic downturns; and (4) simulation, StoApp and GA-based, optimisation tools offer a flexible platform to quantify and compare planning strategies in uncertain environments, and improve performance by optimising design parameters. This research offers insights into the degree of benefits that integrated planning offers in different scenarios; and how different information can be better utilised. The current model does not address establishing higher level of SC cooperation or reducing uncertainty (technologically or otherwise). These are important related topics requiring further research.
